# Update on Berberine in Nonalcoholic Fatty Liver Disease

**DOI:** 10.1155/2013/308134

**Published:** 2013-06-17

**Authors:** Yang Liu, Li Zhang, Haiyan Song, Guang Ji

**Affiliations:** ^1^Institute of Digestive Diseases, Longhua Hospital, Shanghai University of Traditional Chinese Medicine, Shanghai 200032, China; ^2^E-Institute of Shanghai Municipal Education Commission, Shanghai University of Traditional Chinese Medicine, Shanghai 201203, China

## Abstract

Berberine (BBR), an active ingredient from nature plants, has demonstrated multiple biological activities and pharmacological effects in a series of metabolic diseases including nonalcoholic fatty liver disease (NAFLD). The recent literature points out that BBR may be a potential drug for NAFLD in both experimental models and clinical trials. This review highlights important discoveries of BBR in this increasing disease and addresses the relevant targets of BBR on NAFLD which links to insulin pathway, adenosine monophosphate-activated protein kinase (AMPK) signaling, gut environment, hepatic lipid transportation, among others. Developing nuanced understanding of the mechanisms will help to optimize more targeted and effective clinical application of BBR for NAFLD.

## 1. Introduction 

As the global waistline continues to expand, metabolic abnormalities including obesity, type 2 diabetes, hypertension, and dyslipidemia, collectively termed the “metabolic syndrome,” are reaching epidemic proportions. The pathogenesis of these disease state is hypothesized to begin with abnormal accumulation of lipids in nonadipose tissues (steatosis), known as nonalcoholic fatty liver disease (NAFLD), a chronic condition that is currently the leading cause of referrals to hepatology clinics. Due to serious adverse effects and the limited effectiveness of currently available pharmacological therapies for “metabolic syndrome,” many research efforts have focused on the development of drugs from natural products. 

**Figure 1:**
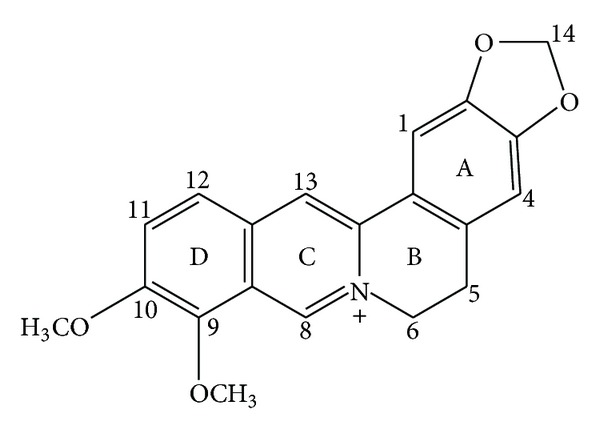
Chemical structure of BBR.

Berberine (BBR, C_20_H_18_NO_4_) is an isoquinoline alkaloid of the protoberberine type, which presents in an array of plants, including Hydrastis canadensis (goldenseal), Coptis chinensis (Coptis or goldenthread), Berberis aquifolium (the Oregon grape), Berberis vulgaris (barberry), and Berberis aristata (tree turmeric) among others. The isoquinoline alkaloid drug belongs to the structural class of protoberberines which includes a quaternary base (Figure 1). There are many derivates and analogues available, such as berberine hydrochloride, berberine sulfate, and berberine citrate or phosphate, contributing to its multiple pharmacological and biochemical effects.

BBR is traditionally used as an antimicrobial and antiprotozoal drug, the antimicrobial activity against a variety of organisms, including bacteria, viruses, fungi, protozoans, helminths, and chlamydia, which have been applied in Chinese medicine for many decades. Recent researches have revealed novel pharmacological properties and multiple therapeutic applications, mainly concerning metabolic diseases, such as obesity and type 2 diabetes [1]. Kinetic study shows that BBR metabolites are widely distributed into various tissues, including liver, heart, kidney, spleen, lung, and even brain, with the liver being the most predominant organ, and average concentration of BBR in liver is approximately 70-fold greater than that in plasma [2]. Other dosing routes, such as femoral vein administration also identified the disposition of BBR in blood, liver, and bile fluid [3]. Additionally, BBR has longer half-life in liver than other tissues [4], suggesting liver as the main target organ of BBR. 

In the past several decades, BBR's action on glucose and lipid metabolic disorders has been widely studied; although the effects or mechanisms of BBR on NAFLD could not separate completely from other metabolic diseases, the role of BBR on NAFLD might be different due to the uniqueness of the organ. This review will mainly focus on BBR on the effect NAFLD and its potential mechanisms.

## 2. Effect of BBR on Fatty Liver

BBR is reported to inhibit cholesterol and triglyceride synthesis in human hepatoma cell line (HepG2) cells and primary hepatocytes [5, 6], and treating rat hepatoma H4IIE cells with BBR shows increased glucose consumption in dose-dependent manner [7]. In vivo data from various animal models also confirm BBR's beneficial role in preventing or treating NAFLD. Intraperitoneal injection of BBR for three weeks has been shown to alleviate hyperlipidemia and fatty liver in obese db/db and ob/ob mice [8]. In Zucker diabetic fatty rats, two-week treatment with a BBR-containing formula could attenuate fatty degeneration [9]. Treatment of hyperlipidemic hamsters with BBR strongly reduces fat storage in the liver [5]. As for mice with high-fat diet (HFD) induced fatty liver, sixteen weeks BBR supplement could alleviate hepatic steatosis and decrease liver lipid content by 14% [10]. This antisteatosis effect of BBR is also reported in diabetic hyperlipidemic rats, which demonstrates that BBR prevents the pathological progression of liver and reverted the increased hepatic triglyceride to near the control levels [11]. In addition, BBR further prevents the development of obesity and insulin resistance in HFD-fed rats [12], and BBR may also prevent liver fibrosis experimental models [13]. Clinical investigations showed that BBR supplement may reduce alanine and aspartate transaminase levels in patients with type 2 diabetes, indicating the restoration of liver function [14, 15]. Furthermore, BBR has been shown to reduce liver necrosis both in nonalcoholic steatosis and in steatosis due to hepatitis C infection [13]. In elderly hypercholesterolemic patients who were previously statin-intolerant, BBR demonstrates reduced cholesterolemia and plasma low density lipoprotein-cholesterol (LDL-c) levels [16].

## 3. Mechanisms of BBR in NAFLD

The precise mechanisms of the development of NAFLD or BBR improving fatty liver remain largely unclear. Defects in lipid metabolism pathways, insulin resistance, and inflammation are crucial players in the process of NAFLD. The recent literature points out that BBR may be integrated into lipid and glucose regulation, combating fatty liver and related syndromes, and the beneficial role of BBR on NAFLD might be achieved through multiple mechanisms.

### 3.1. BBR in Mediating Insulin Resistance

Insulin resistance plays a critical role in the pathogenesis of NAFLD [17]; hence, improving insulin sensitivity is of great importance in dealing with NAFLD. Evidence of BBR on insulin resistance has been elucidated in clinical trials as well as experimental animals and cells lines [14, 18, 19]. Although there is no definitive explanation of how BBR in regulating sensitivity, there are several encouraging observations revealing the possible mechanisms.

After meal, pancreatic islets secrete insulin, and insulin's presence at the cell surface is transduced to cytoplasmic and nuclear responses by tyrosine phosphorylation of insulin receptor substrates (IRSs). Nutrient-induced serine phosphorylation of IRS proteins is proved to be the counter-regulation of the signaling pathway, which blunts insulin action in stressed target tissues and stems the influx of nutrients into already overwhelmed cells [20]. Recently, low grade inflammation and endoplasmic reticulum (ER) stress have been proposed to be in close association with insulin resistance, activation of inflammatory pathways, such as Jun N-terminal kinases (JNKs), inhibitor of nuclear factor B(IkB) kinase*β* (IKK*β*), inositol requiring enzyme 1 (IRE-1), could coverage [21–23]. 

BBR was reported to stimulate insulin secretion in HIT-T15 cells and pancreatic islets, which may have certain influence on its antidiabetic activity [24]. Interleukin-6 (IL-6) and tumor necrosis factor-*α* (TNF-*α*) production in HepG2 cells represent a state of inflammation and consequently impair insulin pathway, and treatment with BBR effectively inhibits IL-6 and TNF*α* production in a concentration-dependent manner, and improves insulin signaling cascade by modification of IRS-1 and Protein kinase B (PKB, Akt) Ser/Thr phosphorylation, indicating the insulin enhacing potential of BBR is through the anti-inflammatory activity [25]. Moreover, pretreating with BBR could block tunicamycin induced ER stress, coexisting with the inhibition of PKR-like eukaryotic initiation factor 2*α* kinase (PERK) and eukaryotic translational initiation factor 2*α* (eIF2*α*) phosphorylation, two protein markers of ER stress, and this results in increasing IRS-1Tyr phosphorylation whereas decreasing its Ser (307) phosphorylation, thus improving insulin resistance under the condition [26]. Other mechanisms, including BBR-caused insulin receptor (InsR) promoter activation and InsR messenger RNA (mRNA) transcription, might also contribute to effect of BBR in regulating insulin sensitivity. In vitro studies further suggest that BBR-induced InsR gene exoression depends on protein kinase C (PKC) activity [27], possibly depends on protein kinase C (PKC) activity in the liver, and BBR mediated PPARs restoration is observed to be in parallel with hepatic glycogen and triglyceride attenuation actions [11]. Our previous work also identified the direct action of BBR on IRS [28]. Taken together, these studies indicate that BBR might be of great insulin sensitive potential and an active player in the liver.

### 3.2. BBR in Regulating APMK Pathway

Adenosine monophosphate-activated protein kinase (AMPK) is an attractive drug target that plays a key role in regulation of whole-body energy homeostasis. Increasing empirical evidence points towards AMPK activation as the target of BBR. In different animal models and various cell lines, phosphorylation of AMPK is identified, attributing to the hypoglycemic and hypolipidemic effect of BBR [5, 7, 29, 30]. To further verify this target, exposure cultured cells with Compound C, an AMPK inhibitor, BBR mediated effect is then abolished [8, 31], implying AMPK as a crucial player of BBR to dissipate stored fat and elevated glucose levels.

The mechanism of BBR on AMPK activation is still elusive, and studies suggest mitochondrial function as the key of this issue. BBR was used as cationic fluorescent probe for investigating energized state of liver mitochondria several decades ago, penetration of BBR into mitochondria could inhibit NAD-linked respiration, and the inhibition is totally dependent on the energization of the membrane [32]. Later, in vitro experiments provided detailed insight into the mechanisms of BBR in regulating this pathway. BBR inhibits glucose oxidation in mitochondria, which leads to an increase in the AMP/ATP (adenosine monophosphate/adenosine triphosphate) ratio in cells, thus accounting for AMPK activation [7, 19, 33, 34]. Other studies further identified complex I inhibition in the mitochondrial respiratory chain as the target of BBR, which blocks AMP conversion into ATP, allowing AMP accumulation in mitochondrial [19, 33, 34]. When elevated AMP binds to the subunit *γ*, the inhibitory domain of the *α*1 subunit is released from the kinase domain, results in an active conformation of AMPK [35]. 

The liver is a vital organ present in vertebrates and has a wide range of functions. Activation of hepatic AMPK leads to increased fatty acid oxidation and simultaneously inhibition of hepatic glucose production as well as lipogenesis and cholesterol synthesis. In vivo data from obese db/db and ob/ob mice indicate that BBR stimulates the expression of fatty acid oxidative genes, while suppresses that of lipogenic genes [8], and BBR also improves insulin resistance in nutrient stressed mice through activation of AMPK [36]. Hepatic sterol regulatory element-binding proteins (SREBPs), liver X receptor *α* (LXR*α*) and peroxisome proliferator-activated receptor *α* (PPAR*α*) transcriptional programs are also observed to be involved in the therapeutic mechanisms of BBR in type 2 diabetic hamsters [37], supporting the concept that BBR prevents dyslipidemia and fatty liver by directly promoting the activation of hepatic AMPK. As an alternative to animal studies, BBR hydrochloride regulates the transcription of hepatic genes that involve in glucose and fatty acid metabolism in vitro experiment using rat primary hepatocytes [6]. More importantly, AMPK specifically binds to and directly phosphorylates SREBP-1c and SREBP-2, and the Ser372 phosphorylation of SREBP-1c by AMPK could inhibit the proteolytic cleavage and nuclear translocation of SREBP-1c in hepatocytes, thereby preventing its autoregulation and transcription of target lipogenic genes [acetyl-CoA carboxylase 1 (ACC1), fatty acid synthase (FAS), and stearoyl CoA desaturase 1 (SCD1)] [38]. Indeed, BBR was reported to inhibit both SREBP1c and SREBP2 expression in hepatocytes [34, 39], quite consistent with its AMPK regulation mechanism, explaining the triglyceride and cholesterol lowering effect of the compound.

### 3.3. BBR in Modifying Gut Microenvironment

Roughly 75% of the blood entering the liver is venous blood from the portal vein, which is all from the digest system. Therefore, the liver gets “first pickings” of everything absorbed in the small intestine. While simple steatosis seems to be well tolerated and to have only mild consequences, a significant proportion of patients with NAFLD develop nonalcoholic steatohepatitis (NASH), a condition that may result in hepatic fibrosis, cirrhosis, and hepatocellular carcinoma [40, 41]. Although the circumstances that may lead to fatty liver progress remain largely unknown, components from the intestinal microflora may contribute to the regulation of proinflammatory processes in the liver that has been investigated in recent years [42].

Due to the low bioavailability and poor absorption through the gut wall, BBR might exert its effect in the intestinal tract before absorption. BBR has been shown to have significant antimicrobial activity against bacteria, fungi, parasites, worms, and viruses. In terms of bacteria, BBR has demonstrated highly significant activity against Staphylococcus, Streptococcus, Salmonella, Klebsiella, Clostridium, Pseudomonas, Proteus, Shigella, Vibrio, and Cryptococcus species [43]. BBR also exhibited effectiveness in combating enterotoxigenic *Escherichia coli* diarrhea [44]. Moreover, BBR inhibited the overgrowth organisms such as staphylococci and coliforms, while having no effect on indigenous lactobacilli and bifidobacteria. 

Alternatively, BBR inhibits a wide range of intestinal microbes, modulates shift of the gut microbiota structure, and enriches some short-chain fatty acid (SCFA) producers in HFD-fed rats [12]. It has been shown that the antimicrobial activity of BBR can be mediated by inhibiting FtsZ (an essential cytoskeleton protein for bacteria cytokinesis) assembly and halting cell division of the bacteria [45]. Additionally, BBR-regulated reversion of inducible cyclooxygenase-2 (COX-2) might also contribute to this process [46]. Dysfunction of gutmicrobiota has more effective capacity to harvest energy from the diet, and when germ-free mice were transplanted with caecal microbiota from ob/ob mice, they developed obesity and insulin resistant within 2 weeks [47], thus the antimicrobial role of BBR might putatively affect energy absorption, which could partially explain its lipid-lowering action under nutrient oversupplying conditions. 

BBR could regulate integrity of tight junction in cultured human Caco-2 cells [48], and similar effect was reflected in a mice model of endotoxemia; pretreatment with BBR attenuates disruption of tight junctions in intestinal epithelium in the animals [49], suggesting BBR's action in reducing epithelial gut permeability under pathogen stressed conditions, with the possible involvement of nuclear factor-*κ*B (NF-*κ*B) and myosin light chain kinase pathways [49, 50]. Therefore, BBR mediated intestinal barrier improvement could block the endotoxemia into circulation, thus reducing hepatic inflammation and preventing NAFLD progression.

### 3.4. BBR on Hepatic Lipid Secretion

Secretion of triglycerides as very low-density lipoprotein (VLDL) is an important aspect in maintaining hepatic lipid homeostasis. Hepatocytes have a unique ability to present triglycerides to the organelles where VLDL assembly takes place. Efficient assembly of apolipoprotein B (apoB) 100 with triglyceride and cholesterol into VLDL requires the activity of an ER-resident microsomal triglyceride transfer protein (MTTP), and loss-of-function mutations within the MTTP gene are the cause of human abetalipoproteinemia, an autosomal recessive disease characterized by the total absence of triglyceride-rich lipoproteins in the plasma [51]. In an HFD-induced NAFLD rat model, Chang et al. [10] observed three increased DNA methylation sites in the MTTP promoter, which account for the reduced MTTP expression in the liver. BBR treatment could cause demethylation of the abnormal regions in MTTP promoter, counteracting HFD-induced MTTP dysregulation. BBR regulated restoration of MTTP expression and VLDL assembly further increase triglyceride secretion and alleviate fatty liver [10].

### 3.5. BBR on Cholesterol Metabolism

BBR has shown considerable impact on cholesterol metabolism, and preclinical and clinical studies both suggest lipid-lowering acting of the drug [52, 53]. The molecular mechanism has been proposed to be through stabilization of the low-density lipoprotein receptor (LDLR) messenger RNA, which led to upregulation of LDLR protein [54, 55]. Other studies illustrate that BBR-induced stabilization of LDLR mRNA is mediated by the extracellular signal-regulated kinase (ERK) signaling pathway through interactions of Cis-regulatory sequences of 3′ untranslated region (UTR) and mRNA binding proteins that are downstream effectors of this signaling cascade [56]. Additionally, SREBPs also act as regulators of hepatic cholesterol levels and activate genes involved in the synthesis of cholesterol and free fatty acids [57]. SREBP cleavage-activating protein has a cholesterol-sensing domain that senses intracellular cholesterol levels and directs the activity of SREBPs. Therefore, the ability of BBR in suppressing SREBPs could be another mechanism for its role in cholesterol metabolism, further explain the beneficial effects on NAFLD.

### 3.6. Other Possible Mechanisms

BBR can downregulate hepatic expression of uncoupling protein-2 (UCP2) mRNA protein in NAFLD rats, promote the recovery of hepatocyte steatosis, and improve lipid metabolism disorder [58]. BBR also demonstrates to have antioxidative activities in cultured cells and animals, and this effect may help reducing reactive oxygen species (ROS) production in the liver [59, 60], thus preventing liver damage. Extrahepatic factors might also affect NAFLD pathology, and in adipocytes, studies illustrate that BBR inhibits adipogenesis in murine-derived 3T3-L1 preadipocytes and human white preadipocytes [61], while enhancing glucose and fatty acid uptake by muscle cells has been proved [62], BBR is proved to play a role in pancreatic islets as well [24]. Both intrahepatic and extrahepatic mechanisms integrate into the BBR regulating action in combating metabolic diseases.

## 4. Prospects of BBR on NAFLD

There is a long history of safe usage of BBR in clinic, sporadic single case reports on the adverse effect of BBR include gastrointestinal side effects, allergic skin reaction and arrhythmia [63]. Even though some reports indicate that BBR could induce hepatoma cells apoptosis, the cytotoxic effects were absent in healthy hepatocytes [64, 65], and BBR actually shows antihepatotoxic action in a series of studies. BBR is investigated in rats with acetaminophen-induced hepatic damage, which shows decreased serum levels of alkaline phosphatase (ALP) and aminotransaminases, suggestive of hepatoprotection of the alkaloid [66]. In cultured rat hepatocytes, Hwang et al. [67] demonstrated bioactivity of BBR in protecting the cells against oxidative damage. 

However, despite the promising effects of BBR on animal models and cells, large clinical investigations are not available. Due to low bioavailability and poor absorption of BBR via oral administration [2], high dose oral administration usually causes gastrointestinal side effects, which greatly limit its clinical application. There are some reports, however, trying to explore new dosage forms of BBR to increase its bioavailability. Preclinical studies that use sodium caprate, one of the intestinal absorption enhancers, showed 1.5- to 2.3-fold increase of BBR bioavailability in different models [68, 69]. P-glycoprotein (P-gp) inhibitors, such as cyclosporine and verapamil, also illustrate marked increase of BBR absorption [70]. Though the beneficial action of BBR is obvious, further studies are still in need to optimize its clinical application in NAFLD. 

## 5. Summary

**Figure 2:**
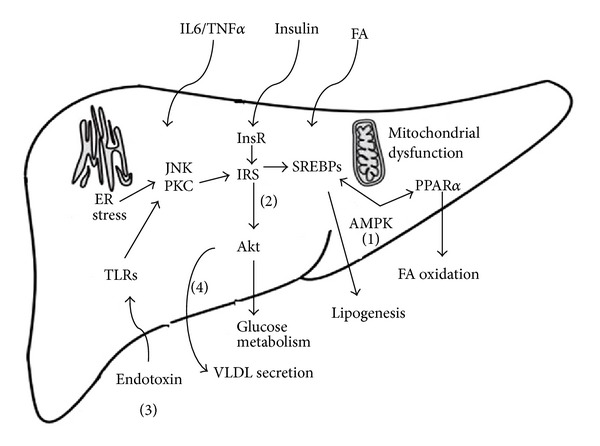
Cellular mechanisms of BBR in reverting dysfunction in NAFLD. Nutrient stress induced a series of alterations in the liver, including mitochondrial dysfunction, ER stress, proinflammatory cytokines and endotoxin elevation, and decreased VLDL secretion. BBR is partitioned toward several pathways in protecting fatty liver. (1) BBR phosphorylates *α* subunit of AMPK through regulating AMP/ATP ratio, and activation of AMPK can inhibit SREBPs to suppress de novo lipogenesis, increasing PPAR*α* expression to enhance fatty acid oxidation in the liver. (2) BBR improves insulin sensitivity by normalize insulin signaling pathway, and BBR reduces pro-inflammatory cytokines production, counteracting ER stress, thus leading to the reviving of insulin signaling transduction. (3) BBR blocks intestinal endotoxin into liver, endotoxin is a major risk factor for NAFLD progression, BBR may mediate gut environment and reduce epithelial gut permeability, which are subsequently avoid the endotoxemia into circulation; (4) BBR promote VLDL secretion by increase ApoB assembly. Additionally, the extrahepatic role of BBR that mediates fatty acid, hormones, and cytokines entering liver also contributes to the lipid-lowering effects of BBR.

NAFLD is the liver manifestation of obesity and the metabolic syndrome and is marked by lipid deposition and/or inflammation. In this review, we introduced the beneficial potential of a nature compound, BBR, in NAFLD and the possible mechanisms under the therapeutic actions (Figure 2). Though most evidence based on experimental studies, and clinical trials need to be further confirmed, we still hold the belief that BBR is a promising candidate in preventing and treating NAFLD in the future. However, more studies should be cautiously performed to clarify the mechanisms and optimize clinical application of the drug.

## Abbreviations

ACC1: Acetyl-CoA carboxylase 1
ALP: Alkaline phosphatase
AMP: Adenosine monophosphate
AMPK: Adenosine monophosphate-activated protein kinase
apoB: Apolipoprotein B
BBR: Berberine
COX-2: Cyclooxygenase-2
eIF2*α*: Eukaryotic translational initiation factor 2*α*
ER: Endoplasmic reticulum
ERK: Extracellular signal-regulated kinase
FAS: Fatty acid synthase
HFD: High-fat diet
IKK*β*: Inhibitor of kappaB kinase *β*
IL-6: Interleukin-6
IRE-1: ER-to-nucleus signaling 1
IRS: Insulin receptor substrate
I*κ*B: Inhibitor of nuclear factor B
JNK: Jun N-terminal kinases
LDL-c: Low-density lipoprotein cholesterol
LDLR: Low-density lipoprotein receptor
LXR*α*: Liver X receptor *α*
mRNA: Messenger RNA
MTTP: Microsomal triglyceride transfer protein
NAFLD: Nonalcoholic fatty liver disease
NASH: Nonalcoholic steatohepatitis
NF-*κ*B: Nuclear factor-*κ*B
PERK: PKR-like eukaryotic initiation factor 2*α* kinase
P-gp: P-glycoprotein
PKB, Akt: Protein kinase B
PKC: Protein kinase C
PPARs: Peroxisome proliferator-activated receptors
ROS: Reactive oxygen species
SCD1: Stearoyl CoA desaturase 1
SCFA: Short-chain fatty acid
Ser/Thr: Serine/threonine
SREBPs: Sterol regulatory element-binding proteins
t-BHP: Tert-butyl hydroperoxide
TNF-*α*: Tumor necrosis factor-*α*
UCP2: Uncoupling protein-2
UTR: Untranslated region
VLDL: Very low-density lipoprotein.
